# The Current Prevalence of Underweight, Overweight, and Obesity Associated with Demographic Factors among Pakistan School-Aged Children and Adolescents—An Empirical Cross-Sectional Study

**DOI:** 10.3390/ijerph191811619

**Published:** 2022-09-15

**Authors:** Moazzam Tanveer, Andreas Hohmann, Nadeem Roy, Asifa Zeba, Umar Tanveer, Maximilian Siener

**Affiliations:** 1 School of Physical Education and Sport Training, Shanghai University of Sport, Shanghai 200438, China; 2 Department of Training and Movement Science, BaySpo—Bayreuth Center of Sport Science, University of Bayreuth, 95447 Bayreuth, Germany; 3 School of Physical Education, Shanxi University, Taiyuan 030006, China; 4 Department of Education, International Islamic University, Islamabad 44000, Pakistan; 5 Department of Mass Communication, University of Lahore, Lahore 54000, Pakistan

**Keywords:** body weight status, demographics, Pakistan, children, adolescents, body mass index

## Abstract

*Purpose:* This study investigated the most recent estimates of underweight, overweight, and obesity prevalence in Pakistani school-aged children and adolescents. *Methods:* A cross-sectional study was conducted using a convenience random clustered sampling approach with 3,551 Pakistani school children aged 9 to 17 years from 52 schools throughout seven districts in central Punjab province. The *CDC US 2000* was used to define underweight (BMI < 5th percentile), overweight (85th ≤ BMI < 95th percentile), and obesity (95th percentile ≤ BMI) for different school grade cohorts (primary, middle, secondary, and higher secondary schools). As a trend test, the Chi-square test was used. A Spearman correlation analysis (*r*) was used to determine the correlations between demographic variables and weight status. A regression analysis was conducted to explore the predictive power of demographic factors in relation to body weight. *Results:* In Pakistani school-aged children and adolescents, the prevalence of underweight, normal weight, overweight, and obesity was 21.9%, 66.9%, 5.8%, and 5.4%, respectively. Significant correlations with body weight status were shown for individual demographic parameters (age, gender, school type, and school grade). Children in urban areas were more likely to be underweight, overweight, or obese than those in rural areas. Boys were found to have a lower BMI than girls. Accordingly, more boys than girls were underweight (odds ratio (OR) = 1.57, 95% CI: 1.33–1.85) and more girls had a higher risk of obesity than boys (OR = 1.39, 95% CI: 1.03–1.86). Lower grades showed more underweight (OR = 1.66, 95% CI: 1.39–1.99) whereas higher grades showed a higher risk of obesity (OR = 1.91, 95% CI: 1.41–2.57). *Conclusions:* In Pakistani school-aged children and adolescents, underweight, overweight, and obesity were prevalent. Compared with studies from 2011, the risk of overweight and obesity in Pakistan has decreased. However, this may also be due to the fact that students in Pakistan have a lower BMI compared to other countries. This issue has also been seen in the present study and is confirmed here by the high number of cases of underweight students. Future research studies should look into additional weight status correlates and factors. To evaluate the association between weight status and behavioral and other health variables, future research should use longitudinal or interventional designs.

## 1. Introduction

Obesity in children is a challenging issue [1]. Because of the harmful effects on children’s health, well-being, and growth, obesity, and physical inactivity in children and adolescents have become global public health issue [2,3]. Obesity in children is one of the world’s most important public health issues in the twenty-first century, affecting every country. Obesity among school-aged children and adolescents has increased by more than tenfold in recent decades [4,5]. Childhood obesity is associated with numerous demographic risk factors [6,7,8]. For example, obesity is associated with an increased risk of developing metabolic syndrome and higher oxidative stress [9,10]. The latter is involved in the development of many diseases, including cardiovascular disease, which is the leading cause of mortality and morbidity worldwide [11,12]. Obesity and insulin resistance are the most important factors in the relationship between metabolic syndrome and oxidative stress and should therefore be quickly identified and treated.

In 2004, the World Health Assembly in Geneva called for quick action to halt the disease, which has spread throughout developing countries, including Pakistan, and primarily affects the wealthier urban population [13,14]. In 2012, 44 million children under the age of five were overweight or obese, accounting for 6.7% of the global population [15]. Around 340 million children and adolescents aged 5 to 19 were overweight or obese in 2016 [16] and according to the World Health Organization, 39 million children under the age of five were overweight or obese in 2020 [17]. Thus, obesity and overweight are on the rise in low- and middle-income countries, especially in urban areas, where they have long been regarded as problems exclusively in high-income countries [18]. Increases in body mass index (BMI) in rural areas, on the other hand, accounted for more than 55% of the global increase in mean BMI between 1985 and 2017—and more than 80% in some low- and middle-income regions [19,20]. Obesity-related ailments are more prone to strike them [21].

Obesity is growing more common in all countries [22]. Pakistan (~220 million inhabitants) is a low- and middle-income country with 54 percent of the population under 19 years old. The country has been ranked 92nd out of 116 countries in the global hunger index for 2021, with a serious hunger problem [23]. In terms of obesity, Pakistan ranks tenth out of 188 countries, with 50% of the population being overweight or obese. According to the World Obesity Federation, 5.4 million Pakistani school-aged children will be obese by 2030 [24]. Thus, Pakistan is grappling with the twin problems of over-nutrition and poor nutrition. The WHO Diabetes country profiles show that Pakistan has yet to implement an operational policy to combat overweight and obesity.

While there are already some studies on the BMI of children and adolescents in China [2,3,25], Italy [8,26], America [27,28,29], Brazil [30], India [31], Saudi Arabia [32], Cyprus [33], and Poland [34], very limited data have been available from Pakistan so far. This is surprising since Pakistan is one of the five most populous countries in the world, with more than 220 million inhabitants. Mushtaq et al. [13] published the only study on Pakistani primary school-aged children; this was more than 10 years ago. It is precisely due to the fact that there is limited research that there is still a gap regarding the BMI of Pakistani schoolchildren [35]. Therefore, from a public health perspective, it is critical to collect up-to-date population-based surveillance data on school-aged children to plan and develop timely health promotion activities and interventions in schools and communities [2,13].

Therefore, the aim of this study is to obtain an up-to-date picture of the prevalence of underweight, normal weight, overweight, and obesity among Pakistani schoolchildren and to determine which socio-demographic factors are associated with the occurrence of body weight risks. The results can later be used to determine which groups of people are particularly at risk and are candidates for a possible health intervention.

## 2. Materials and Methods

### 2.1. Study Design, Setting and Participants

A cross-sectional study was conducted in the summer of 2021 among school-aged children and adolescents. Children aged 9–11 years old and adolescents aged 12–17 years old [27] were sampled from primary (grades 4–5), middle (grades 6–8), secondary (grades 9–10), and higher secondary schools (grades 11–12). The division of school grades is based on the Pakistani school system. Children usually start elementary school at the age of five, and depending on their school performance, move on to subsequent school types (middle school: 10–13 years old; secondary school: 13–15 years old; higher secondary school: 15–17 years old). Primary school grades 1st to 3rd were excluded from this study due to concerns regarding their cognitive capacity to complete the survey [2].

In this study, a convenience sample with random clustered sampling was used [36,37]. A total of 3551 students from 52 schools volunteered for this study. One class from each grade level from each participating school was included in the study. The Punjab school education department (https://sis.punjab.gov.pk/, accessed on 23 December 2019) has compiled a comprehensive list of schools in the Punjab region. This overall school list included administrative cities and districts, smaller towns, and municipal/local districts, which represent a mix of rural and urban areas. Based on this list, the participating schools were randomly selected from a total of seven districts (Lahore, Gujranwala, Gujrat, Sheikhupura, Narowal, Hafizabad, and Sialkot) of the central Punjab region. The seven districts selected included purely urban areas, such as Punjab’s capital Lahore (11 million inhabitants), and predominantly rural areas, such as the district of Narowal (urbanization: 15%). The Punjab region, with 110 million inhabitants, is one of the most populous regions in Pakistan. However, due to the huge number of inhabitants and the fact that a large part of Pakistan’s industrial production is located there—which, in turn, also has an impact on government subsidies—Punjab can be described as one of the most prosperous regions compared to other regions of Pakistan. The literacy rate in most of the selected districts is over 70%. Because Punjab represents about 50% of Pakistan’s population, and our study drew a random sample from schools in this region, it can be assumed that the participants represent a good picture of all Pakistani students. Nonetheless, it can be expected that the situation in other regions may differ from the situation in Punjab, especially in smaller, financially weaker regions with low literacy rates and small populations. However, this would affect only a small part of the Pakistani population.

The Shanghai University of Sport Institutional Ethics Committee authorized the study [1816111009-2019], and permission to conduct the study was acquired from the participating schools’ teachers and principals. There was also a letter of approval from the Punjab School Education Department to conduct the study. All the children and adolescents in the study, as well as their parents or guardians, were told that participation was entirely voluntary. Before data collection, all parents or guardians gave their verbal informed consent, and all children gave their verbal positive assent. The data were collected and processed anonymously.

### 2.2. Measurements

Trained rescue professionals from the Punjab Emergency Service Department collected anthropometric measurements of weight and height in the classroom. The questionnaire was written in English and read aloud to children in lower grades with translation in Urdu if required. The students’ body weights and heights were measured first. Afterwards, the students completed the survey questionnaire on paper in a classroom setting.

### 2.3. Weight Status

Body weight was measured to the nearest 0.1 kg and body height to the nearest 0.5 cm [2,13,38]. The digital CERTEZA weight machine was used to measure body weight. A SECA scale (https://www.seca.com/patents, accessed on 14 September 2021) was used to measure the student’s body height. Body height and weight were taken from students wearing light summer school uniforms without shoes and without a cap with a correct standing position during the early mornings or early in the afternoons [13].

### 2.4. Demographic Factors

A paper questionnaire, which had to be completed by the students themselves, was used to collect demographic data. The questionnaire contained demographic information on school type (public and private schools), gender (boy or girl), age (9 to 17 years), age category (child or adolescence), school level (primary school, middle school, secondary school, or higher secondary school), religion (Muslim or non-Muslim), and place of residence (urban or rural). 

The test–retest reliability shows a Cronbach’s alpha of 0.84 [39,40,41]. Demographic data were chosen based on the study by Mushtaq et al. [13] who conducted a similar survey in Pakistan in 2011.

### 2.5. Statistical Analysis

IBM SPSSv.26 Statistical Analysis was used to analyze the data. Statistical significance was determined using *p* ≤ 0.05.

BMI was calculated by dividing a person’s weight in kilograms by their height in meters squared (kg/m^2^). The body weight status of each participant was determined using the CDC US 2000 BMI charts [38,42]: Underweight (BMI < 5th percentile), normal weight (5th percentile ≤ BMI < 85th percentile), overweight (85th percentile ≤ BMI < 95th percentile), and obesity (95th percentile ≤ BMI).

A frequency distribution analysis was performed to show the prevalence of underweight, normal weight, overweight, and obesity within the sample.

Chi-square tests were calculated using body weight status as the dependent variable and each demographic factor (e.g., sex, age, residence, school type, school grade, or religion) as the respective independent variable [13,43,44]. As a result, it can be determined, for example, whether boys and girls show significant differences in body weight status. In addition, a correlation analysis was used to measure the relationship between body weight status and demographic factors.

Linear regression analysis was used to explore the predictive power of demographic parameters (independent variables) on body weight status (dependent variable) [13].

To determine the effect of demographic factors on the occurrence of underweight, overweight, and obesity, three binary logistic regression analyses were performed [2,43,45]. For each of these three regression analyses, a new dependent variable was created in which the body weight status sought (e.g., underweight) was coded 1 and all other body weight groups were coded 0. Subsequently, the explanatory power of the demographic factors (independent variables) with respect to this newly created dependent binary variable (e.g., underweight: Yes = 1, No = 0) was calculated, and odds ratios (OR) were computed.

To gain a more detailed insight into which of the four possible school levels has a direct influence on the individual body weight status results, four new binary variables were determined. Each of these binary variables was coded 1 if the school level (e.g., elementary school) applied to the child and coded 0 if it did not. Using these new variables, the OR of each individual school level on the occurrence of a body weight problem was also calculated. This procedure allows a better estimation of which school level (elementary school, middle school, secondary school, or higher secondary school) is responsible for significant effects in the overall variable school levels.

From the corresponding value of the calculated OR, it can be determined how strongly the influence of the demographic factor is on the risk of belonging to the searched body weight status group. ORs take on values greater than zero, with a value of 1 having no effect on the dependent variable. The more the value deviates from 1, the greater the respective effect can be assumed.

## 3. Results

### 3.1. Study Participants

In the study, 3551 out of a total of 3600 students (=100%) tested had completed their questionnaires—that was, 98.6%. A total of 49 students, or 1.4%, were disqualified due to missing or insufficient information. 

The sample sizes and demographic information for all four levels of schools are presented in Table 1. The percentage of male participants was 54.4%, and 45.6% were female participants. Children (aged 9 to 11 years) accounted for 20%, and adolescents (aged 12 to 17 years) accounted for 80%. A total of 96.3% of the students were Muslim, and 3.7% were non-Muslim. The students were 54% from urban areas and 46% from rural areas. The students were 75.4% from private schools and 24.6% from private schools.

### 3.2. Prevalence of Body Weight Status

**Figure 1:**
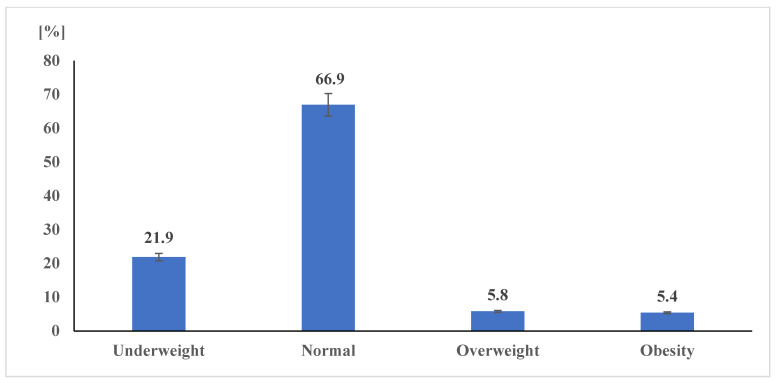
Descriptive statistics for the current prevalence of body weight status (*N* = 3551; underweight *n* = 778, normal weight *n* = 2377, overweight *n* = 205, obese *n* = 191).

The current prevalence of body weight status among Pakistani school-aged children and adolescents is depicted in Figure 1. The overall prevalence of underweight, normal weight, overweight, and obesity was found to be: underweight 21.9%, normal weight 66.9%, overweight 5.8%, and obesity 5.4%.

### 3.3. Correlation of Demographic Parameters with Body Weight Status

The bivariate correlations are shown in Table 2. Body weight status and gender were significantly positively related (*r* = 0.093 **). Body weight status also had a significant positive correlation with age (*r* = 0.066 **); school type (*r* = 0.054 **), and school level (*r* = 0.110 **). Nevertheless, there was no significant correlation between religion (*r* = −0.014) and residency (*r* = −0.004) and body weight status. Overall, only weak correlations between the parameters were demonstrated.

### 3.4. Differences in Body Weight Outcomes Due to Demographic Factors

The distribution of demographic factors within the four possible weight groups is shown in Table 3. The prevalence of underweight problems was significantly greater among boys (25.4%) than girls (17.8%). Nevertheless, the prevalence of overweight and obesity was greater among girls (12.7%) than boys (9.9%). Children have more underweight problems than adolescents (23.2% vs. 21.6%). However, adolescents were more often overweight and obese than children (12.1% vs. 7.4%). In terms of the underweight problem, non-Muslim students (25.0%) were more affected than Muslim students (21.8%). Among Muslim students, 11.2% were overweight and obese, compared to 9.8% among non-Muslim students. Overall, students from urban areas had higher weight status problems than did participants from rural locations. The prevalence of the underweight problem was also higher among public schools (22.8%) than in private schools (19.1%). The prevalence of overweight and obesity was higher in private schools (14.0%) than in public schools (10.2%). Underweight problems were greater at the primary schools’ level (29.1%) than at the other levels of schools. Overweight and obesity problems were greater at the secondary school level than in the other grades.

In the chi-square test, the factors of gender, age category, place of residence, school tpe, and school level significantly affected the four possible weight groups (underweight, normal weight, overweight, and obesity). In contrast, no significant correlation was demonstrated between body weight status and religion (*p* = 0.066).

A comparison of the sex-specific prevalence of body weight status with demographic parameters is shown in Table 4. At ages 9 to 11, boys and girls are equally affected by being underweight. The incidence of being underweight was about 23% in both groups. However, as the students get older, the figures shift significantly with regard to underweight students, with a strong disadvantage for boys. Boys, at almost 26%, were about 9% more likely to be underweight than girls at this age. The same significant trends were seen for boys and girls in Muslim children. For non-Muslim pupils, the frequency of suffering from being underweight increases once again. Accordingly, 27% of non-Muslim boys were affected by being underweight, and 22% of girls. However, the subsample of non-Muslim students was too small to demonstrate a significant difference between boys and girls. Additionally, related to place of residence, significant differences were found between boys and girls regarding their body weight status. For example, boys from urban public primary schools are statistically the largest risk group for being underweight.

Overweight and obesity were far less common among Pakistani boys and girls than BMI percentiles would suggest. Only private school boys and secondary to higher secondary school girls had slightly higher than average percentages (>15%). Looking at the percentages across all factors, no clear trend could otherwise be identified. For example, public school girls are more likely to be overweight than boys. In private schools, however, the picture changes. Here, boys are more likely to be overweight than girls. The same applies to Muslim and non-Muslim children and primary and middle school students.

The results of the chi-square test show that the body weight status calculated has significant differences for most demographic factors with respect to gender. Only for the factor of children, students of non-Muslim religion, and students of private schools could no significant differences be found with respect to gender in the three body weight groups.

The results of the linear regression analysis showed that three of the six demographic factors had a significant effect on body weight status. The data indicate that sex (*β* = 0.085, SE = 0.08, *p* ≤ 0.001), school type (*β* = 0.054, SE = 0.05, *p* = 0.001), and school level (*β* = 0.167, SE = 0.02, *p* ≤ 0.001) had a positive significant effect on body weight status. Estimates of the model parameters (both unstandardized and standardized) for body weight status are shown in Table 5. The variables of age, religion, and place of residence do not appear to have a significant effect. The whole analysis reached an *R^2^* value of 0.025, with *F*(6, 3544) = 14.379 and *p* ≤ 0.001. If the same calculations are made for BMI, the explained variant increases to about 20% (*R*^2^ = 0.202).

The results of the three binary logistic regressions are shown in Table 6. The first logistic regression examined the association of demographic variables with underweight (Yes = 1, No = 0). As expected, the four variables of gender, residence, school type, and school level show significant results for the risk of being underweight. With an OR of 1.57 (95% Cl: 1.33–1.85), gender had one of the highest influences (boys were coded 1, girls 0). As a second important parameter with an OR of 0.70 (95% Cl: 0.60–0.82) the school level could be determined as a significant factor. If we look at this variable more closely and recode the individual school types into new binary variables, e.g., elementary school = 1, and all other school types = 0, the effect of the individual subgroups within the regression analysis can be estimated even better. If we proceed in this way and calculate new binary regression analyses for the four new binary variables (the respective school level is coded as 1 and all other school levels are coded as 0), it can be seen that only the variables primary school (*p* < 0.001) and secondary school (*p* ≤ 0.01) achieve significant results. The variable primary school reached an OR of 1.66, and secondary school reached 0.78. Accordingly, the risk of suffering from underweight is 66% higher for primary school students than for students at other school levels. In addition to school level, the two variables school type (OR = 0.8, *p* ≤ 0.05; coding: Public = 0 and Private = 1) and residence (OR = 1.2, *p* ≤ 0.05; coding: Rural = 0 and Urban = 1) also reach significant values.

In the second binary logistic regression, the chance of being overweight was calculated. The results show that only the variable of religious affiliation has a significant influence on the risk of being overweight. Accordingly, the odds ratio of being overweight is four times higher for Muslim children than for non-Muslim children. However, the sample of non-Muslim children is very small (*n* = 2, see Table 3).

The last binary logistic regression considers the influence of the demographic factors of being obese. Comparable to the results for being underweight, four factors again show significant results. However, in contrast to underweight students, the variable school type is insignificant. On the other hand, age (OR = 2,01; *p* ≤ 0.01) now shows a significant influence on the dependent variable. The odds ratio of suffering from obesity is, therefore, twice as high for older students as for younger students. The risk of being obese is also higher for girls than for boys by a factor of OR = 1.39 (95% Cl: 1.03–1.86). Analogous to underweight, the school level becomes significant here as well. If the (binary) school level comparison is also determined here, it becomes apparent that, as before, only the primary and secondary school levels differ significantly from the other school levels. The risk of obesity is therefore only half as high for primary school students (OR = 0.43; *p* ≤ 0.01) as for students at other school levels. In contrast, secondary school students are significantly more affected by obesity (OR = 1.91; *p* ≤ 0.01) than students of other school levels. The result for the higher secondary school level is not significant.

## 4. Discussion

In the present study, the prevalence of underweight, normal weight, overweight, and obesity among Pakistani students was determined, and the impact of various demographic factors on the four body weight status groups was calculated.

According to current estimates, 21.9% (25.4% boys and 17.8% girls) of Pakistani school-aged children and adolescents were underweight, 66.9% normal weight (64.7% boys and 69.6% girls), 5.8% overweight (5.3% boys and 6.4% girls), and 5.4% were obese (4.6% boys and 6.3% girls). A study by Mushtaq et al. [13] conducted in 2011 in Lahore (Pakistan) with 1860 primary school children showed that 17% of children aged 5 to 12 were overweight, and 7.5% were obese. In another study of 500 Pakistani high school students in 2013 in Hyderabad [41], 12% were obese, and 8% were overweight. In 2018, a local study was conducted on children aged 3 to 18 years in Multan [46], the results showed that 10% of the students were overweight and 5% were obese. The percentage of overweight students found in previous studies was higher than the results we found. Nevertheless, the number of obese students is somewhat lower than in our study. According to a report published by the World Obesity Federation in 2018, an estimated 6.6% of Pakistani children aged 10–19 were obese, and 10.7% were overweight [24]. Compared with previous studies in Pakistan, our study showed a low percentage of overweight and obesity. The differences in the results could be due to the subject groups. The studies mentioned above were mainly conducted in urban areas or examined in smaller samples of a specific age group. According to the literature, BMI correlates also differ depending on location [3,25,38]. Therefore, the differences in the frequencies could also be due to the fact that, as a result of the widespread rural exodus, the poorer suburban districts in particular have received a strong influx, and the new population group is shifting the previous data in the direction of underweight. However, this assumption must first be verified by further studies. 

The risk of students suffering from underweight was very high in Pakistan. For example, the results show that every fourth boy of school age is underweight. Similar trends were also observed in studies conducted by Mushtaq et al. (2012) [47], They compared the BMI of children from Pakistan with the WHO and US CDC 2000 BMI benchmark tables, showing that the BMI of children from Pakistan is far below the reference values. These trends are also confirmed in the present results. However, the risk of underweight was significantly higher than previously suspected. The problem of being underweight becomes even more evident when the numbers are compared with data from China. While 20% of students in Pakistan are underweight, Chinese students account for only 2.1% [2]. This shows the tremendous economic, social, and educational differences between these Asian countries.

Our study found a significant correlation between weight status and type of school. In contrast, in 2013, a study conducted in the Hyderabad urban region [41] showed no significant correlation between weight and school type. In a different study, urban areas in Karachi were used to recruit 887 school children between the ages of 11 and 15. The results showed a significant correlation between school type, overweight (19.1%), and obesity (10.8%). That study also showed a high percentage of obesity in private schools and a low percentage in public schools [35]. Another local study was conducted in Lahore urban areas from two private schools with 293 students. They found that 11.9% of students in private schools in grades 6 and 7 were obese, while 21.8% were overweight [48]. Their study also showed a high percentage of overweight and obesity among middle school level students. Overall, 5.5% of all middle school students were overweight, and about 4.9% were obese.

Duan et al. [25] conducted a survey of Chinese middle school students and distinguished the results in terms of geographic location. They reported that students in suburban schools engage in less physical activity than students in urban schools. Their study also showed higher weight problems among the urban areas’ students. Furthermore, Zhu et al. [3] investigated geographic differences in children who failed to meet fitness standards. Accordingly, the effects of urbanization on obesity that have been documented elsewhere [2,38] also appear to be evident in China and Pakistan. Thus, the results of the aforementioned studies are in agreement with our findings. In addition, our results showed that students from urban areas were more affected by overweight and obesity than those from rural areas.

According to a study conducted by Ogden et al. [27], in the US, 15.5% of adolescents and 15.3% of children were overweight. Compared to their study, the results of the present study were approximately the same, as overweight problems in Pakistan are also slightly higher in adolescents than in children. However, the prevalence of overweight and obesity was low in Pakistani children and adolescents compared with US children and adolescents. Since a higher school level is reached with increasing age, it can be assumed that the risk of being overweight also increases with a higher school level. Further, a study conducted by Davies et al. [28], showed that a higher level of education or schooling has positive direct effects on physical activity, while it has negative direct effects on BMI and sedentary behavior. Ickovics et al. [29] found that an increase in the BMI of students was also influenced by their school environment. Since more than half of the students in this study were already overweight or obese, school systems should consider earlier interventions. In Pakistan, according to the results of the present study, it looks as if higher grades of schooling correlate with a higher risk of obesity. However, the extent to which Pakistani schooling and age effects are mutually dependent remains to be clarified in future studies.

Dos Santos et al. [30] investigated Brazilian schoolchildren and adolescents and found that different weight groups had different values for the predictor variables. While the results of their logistic regression showed that birth weight had no significant influence on weight status, sex, and age appeared to be significant predictors of children’s weight status. This study found that age and sex were significant predictors of weight status in Pakistani school-aged children and adolescents. According to our study, boys were more often underweight than girls. In a study on Polish school children and adolescents, Czyż et al. [34] discovered that girls were more often underweight than boys. Their study showed that for both gender and age categories, overweight, and obesity indices showed a significant correlation for adolescence. However, their study also showed that sex had no significant effects on body weight at the age of 9–11. They suggested aerobic exercise as a way for physical education instructors, parents, and children to reduce the risk of being overweight and obese. 

This study on school-aged children and adolescents in Pakistan showed a significant association between sex and body weight. Children in Canada also had the same outcomes, according to Ames et al. [49]. Additionally, a study among Indian school-age children [50], and a previous study among school children in Sylhet, Bangladesh [51], their studies showed similar results regarding weight status and sex. Body weight and sex had no significant association, according to two studies conducted among schoolchildren in urban Ghanaian districts [52] and Karachi, Pakistan [35]. Our study on school-aged children and adolescents in Pakistan found that female students were more likely than male students to be overweight and obese. In addition, a study from Hyderabad [41] showed that in secondary schools, girls were more often overweight and obese than boys. Nevertheless, similar studies from different countries revealed that male students had higher rates of obesity and overweight than female students [53,54,55,56].

Using the demographic correlations between body weight status and Pakistani school children and adolescents presented in Table 3, it can be shown that in the results presented, there is also a significant relationship between body weight and sex, age, residence, school type, and school level, but no significant relationship with religion.

Pelusi et al. [8] investigated the correlations between adolescent behavior and demographic variables in Italy and showed that adolescent obesity is influenced by demographic and educational variables. According to Gray et al. [7], demographic factors were the most closely connected to school children and adolescents’ body weight. However, their study involved a sample of American children whose lifestyles differ from those of Pakistan’s traditional Muslim society, according to Ali et al. [57]. due to a lack of institutes, underqualified staff, outdated equipment, and a lack of social awareness, whereas the situation in the United States is the polar opposite.

The current study leads to a better understanding of Pakistan’s health challenges, as well as a more significant understanding of global health trends and the factors affecting these situations. One of the study’s strengths is that it provides actual data on Pakistani school children and adolescents. Demographics and body weight correlates can be used to support, refute, or improve existing theories about students’ body weight issues in school children and adolescents. The current study is a comprehensive analysis of various demographic characteristics. The only study found so far with a comparable number of demographic factors and subjects from Pakistan was by Mushtaq et al. [13] from Lahore in 2011, but their study only examined primary school students. All other studies from Pakistan are limited to smaller or selected samples that include only certain age groups, school types, or residences [35,41,46].

First, the sampling technique of our study comprised selecting administrative cities and districts, towns, and local community districts that represented a mix of rural and urban areas, which could lead to a broader generalization of the results for Pakistan. Second, our study was the first to look at the relationship between weight status and a variety of significant factors (gender, residence, religion, school type, and school level) in Pakistan, all of which are critical in designing community- and family-based policies and interventions. Our study’s nonsignificant findings across various demographic variables indicate the necessity for a more finely grained analysis of children’s BMI category that considers interactions. Future research on associated effective interventions and prevention would require the data provided by this study as a foundation for any practical action, and any future programming shown to be useful in Pakistan could be taken for use in other nations.

The current study has some major limitations. First, we obtained students’ weight status by calculating BMI, which is weight in kg divided by height in meters square, and then used the CDC US 2000 BMI chart for both boys and girls [42]. However, this may not represent an accurate measure of body fat percentage. The number of overweight children could drop slightly among older students by adjusting for body fat. Second, the exclusion of students in Grades 1 through 3 from primary schools may have limited the generalizability of the prevalence estimates to students in primary schools. Finally, because the current study used a cross-sectional approach, no judgments concerning causality could be drawn. To investigate the causal relationship, studies with longitudinal and experimental designs are necessary. In our study, only a few factors influencing the weight status of Pakistani children and adolescents were examined; several other relevant influencing factors (e.g., sleep, food, and unhealthy diet) were not considered. Future studies should therefore address these parameters and investigate them further.

## 5. Conclusions

The findings show that underweight, overweight, and obesity are prevalent among Pakistani school-aged children and adolescents, which is a serious problem. One in five students is affected by being underweight. Furthermore, body weight status had a significant relationship with sex, age, residence, school type, and school level. Girls were more likely to be overweight or obese than boys, while boys were more likely to be underweight. The percentages of underweight, overweight, and obese individuals in the sample changed over age. Thus, higher rates of overweight and obesity were found in adolescents than in children, whereas children were increasingly underweight than older students. According to the study, the risk of being underweight is highest for urban boys attending public primary schools. In contrast, obesity most affected higher secondary school girls in urban environments.

Based on the results, the task now is to create appropriate interventions, especially for high-risk groups, and improve the health of Pakistan’s students. To evaluate the success of these interventions, anthropometric measurements should be taken in all schools at least once a year. Future research should also use longitudinal or interventional designs to determine the relationship between underweight, overweight, and obese with behavioral and other health indicators.

## Figures and Tables

**Table 1:** Descriptive statistics of demographic characteristics.

Variable	Primary School	Middle School	Secondary School	Higher Secondary School
**Sample size, *n* (%)**	797 (22.4)	1368 (38.5)	954 (26.9)	432 (12.2)
**Age (year; mean ± SD)**	10.90 ± 1.23	13.13 ± 1.36	15.29 ± 1.11	16.81 ± 0.44
**Sex, *n* (%)**				
Boys	434 (54.5)	704 (51.5)	558 (58.5)	235 (54.5)
Girls	363 (45.5)	664 (48.5)	396 (41.5)	197 (45.6)
**Age category, *n* (%)**				
Children 9–11 years	567 (71.1)	151 (11.0)	1 (0.1)	0 (0)
Adolescents 12–17 years	230 (28.9)	1217 (89.2)	953 (99.9)	432 (100)
**Religion, *n* (%)**				
Muslims	756 (94.9)	1316 (96.2)	921 (96.5)	426 (98.6)
Non-Muslims	41 (5.1)	52 (3.8)	33 (3.5)	6 (1.4)
**Residence, *n* (%)**				
Urban	616 (77.3)	658 (48.1)	493 (51.7)	155 (35.9)
Rural	181 (22.7)	710 (51.9)	461 (48.3)	277 (64.1)
**School type, *n* (%)**				
Public	606 (76.0)	986 (72.1)	665 (69.7)	421 (97.5)
Private	191 (24.0)	382 (27.9)	289 (30.3)	11 (2.5)
**BMI (mean ± SD)**	15.96 ± 2.52	17.88 ± 3.36	19.57 ± 3.87	20.40 ± 3.53

Legend: BMI = body mass index.

**Table 2:** Correlation coefficient between body weight status and demographic characteristics.

Variable		1	2	3	4	5	6	7
**1**	**Body weight status**	—						
**2**	**Sex**	0.093 **	—					
**3**	**Age**	0.066 **	−0.071 **	—				
**4**	**Religion**	−0.014	−0.004	−0.012	—			
**5**	**Residence**	−0.004	−0.046 **	0.174 **	0.040 *	—		
**6**	**School Type**	0.054 **	−0.008	−0.129 **	0.026	−0.098 **	—	
**7**	**School Level**	0.110 **	−0.026	0.855 **	−0.051 **	0.216 **	−0.072 **	—

Legend: *N* = 3551; small effect for 0.1 ≤ *r* ≤ 0.3; * *p* ≤ 0.05, ** *p* ≤ 0.01.

**Table 3:** Chi-square test (2-tailed) to compare the prevalence of body weight status with demographic parameters (sex, age, residence, religion, school type, and school level).

		*Body Mass Index*				
	Underweight	Normal Weight	Overweight	Obesity ***n (%)***		
Variables	***n (%)***	***n (%)***	***n (%)***		χ^2^	***p*** **-Value**
**Sex**						
Boys	490 (25.4)	1250 (64.7)	102 (5.3)	89 (4.6)	32.71	0.001
Girls	288 (17.8)	1127 (69.6)	103 (6.4)	102 (6.3)		
**Age category**						
Children 9–11 years	167 (23.2)	499 (69.4)	31 (4.3)	22 (3.1)	13.88	0.003
Adolescent 12–17 years	611 (21.6)	1878 (66.3)	174 (6.1)	169 (6.0)		
**Religion**						
Muslim	745 (21.8)	2291 (67.0)	203 (5.9)	180 (5.3)	7.19	0.066
Non-Muslim	33 (25.0)	86 (65.2)	2 (1.5)	11 (8.3)		
**Residence**						
Urban	449 (23.4)	1232 (64.1)	121 (6.3)	120 (6.2)	16.88	0.001
Rural	329 (20.2)	1145 (70.3)	84 (5.2)	71 (4.4)		
**School Type**						
Public	611 (22.8)	1793 (67.0)	132 (4.9)	142 (5.3)	17.64	0.001
Private	167 (19.1)	584 (66.9)	73 (8.4)	49 (5.6)		
**School Level**						
Primary	232 (29.1)	511 (64.1)	31 (3.9)	23 (2.9)	59.85	0.001
Middle	284 (20.8)	942 (68.9)	75 (5.5)	67 (4.9)		
Secondary	181 (19.0)	630 (66.0)	66 (6.9)	77 (8.1)		
Higher Secondary	81 (18.8)	294 (68.1)	33 (7.6)	24 (5.6)		
**Total**	778 (21.9)	2377 (66.9)	205 (5.8)	191 (5.4)		

**Table 4:** Chi-square test to compare the sex-specific prevalence of body weight status with demographic parameters (age, residence, religion, school type, and school level).

		*Body Mass Index*				
		Underweight	Normal Weight	Overweight and Obese		
Variables	Sex	***n (%)***	***n (%)***	***n (%)***	χ^2^	***p*** **-Value**
**Age category**						
Children 9–11 years	Boys	80 (23.6)	229 (67.6)	30 (8.8)	2.25	0.324
	Girls	87 (22.9)	270 (71.1)	23 (6.1)		
Adolescent 12–17 years	Boys	410 (25.8)	1021 (64.1)	161 (10.1)	44.02	0.001
	Girls	201 (16.2)	857 (69.1)	182 (14.7)		
**Religion**						
Muslim	Boys	470 (25.3)	1205 (64.9)	183 (9.8)	32.42	0.001
	Girls	275 (17.6)	1086 (69.6)	200 (12.8)		
Non-Muslim	Boys	20 (27.4)	45 (61.6)	8 (11.0)	0.88	0.641
	Girls	13 (22.0)	41 (69.5)	5 (8.5)		
**Residence**						
Urban	Boys	285 (28.4)	604 (60.1)	116 (11.5)	29.44	0.001
	Girls	164 (17.9)	628 (68.5)	125 (13.6)		
Rural	Boys	205 (22.1)	646 (69.8)	75 (8.1)	8.61	0.014
	Girls	124 (17.6)	499 (71.0)	80 (11.4)		
**School Type**						
Public	Boys	388 (26.8)	944 (65.1)	118 (8.1)	36.71	0.001
	Girls	223 (18.2)	849 (69.1)	156 (12.7)		
Private	Boys	102 (21.2)	306 (63.6)	73 (15.2)	5.24	0.073
	Girls	65 (16.6)	278 (70.9)	49 (12.5)		
**School Level**						
Primary	Boys	143 (32.9)	260 (59.9)	31 (7.1)	7.64	0.022
	Girls	89 (24.5)	251 (69.1)	23 (6.3)		
Middle	Boys	170 (24.1)	473 (67.2)	61 (8.7)	12.71	0.002
	Girls	114 (17.2)	469 (70.6)	81 (12.2)		
Secondary	Boys	131 (23.5)	350 (62.7)	77 (13.8)	17.87	0.001
	Girls	50 (12.6)	280 (70.7)	66 (16.7)		
Higher Secondary	Boys	46 (19.6)	167 (71.1)	22 (9.4)	6.60	0.037
	Girls	35 (17.8)	127 (64.5)	35 (17.8)		
**Total**		778 (21.9)	2377 (66.9)	396 (11.2)		

**Table 5:** Linear regression analysis of demographic factors (independent variables) and body weight status (dependent variable).

		Unstandardized Coefficients		Standardized Coefficients		
	Variables	B	SE	*Β*	*T*	Sig.
	Constant	1.634	0.130		12.542	0.001
**1**	Sex	0.120	0.023	0.085	5.103	0.001
**2**	Age	−0.014	0.010	−0.046	−1.434	0.152
**3**	Religion	0.015	0.062	0.004	0.238	0.812
**4**	Residence	−0.042	0.024	−0.030	−1.747	0.081
**5**	School Type	0.087	0.027	0.054	3.197	0.001
**6**	School Level	0.117	0.024	0.158	4.954	0.001

Abbreviations: SE = standard error.

**Table 6:** Odds ratios of three (binary) logistic regression analyses for the association of underweight, overweight, and obesity with different demographic factors.

	Underweight		Overweight		Obesity	
Variables	OR	95% CI	OR	95% CI	OR	95% CI
**Sex ^a^**	1.57 ^†^	1.33–1.85	0.82	0.61–1.08	0.71 *	0.53–0.96
**Age category ^b^**	0.90	0.74–1.10	1.45	0.98–2.14	2.01 ^‡^	1.27–3.16
**Religions ^c^**	1.19	0.80–1.78	0.24 *	0.06–0.92	1.63	0.86–3.08
**Residence ^d^**	1.20 *	1.02–1.41	1.23	0.92–1.64	1.46 *	1.08–1.97
**School type ^e^**	0.80 *	0.66–0.96	1.76	1.30–2.36	1.06	0.76–1.48
**School level**	0.70 ^†^	0.60–0.82	1.18	0.90–1.54	1.37 *	1.05–1.79

Significance level: ^†^ *p* ≤ 0.001, ^‡^ *p* ≤ 0.01, * *p* ≤ 0.05; Abbreviations: CI = confidence interval, OR = Odds Ratio; Coding: ^a^ Girls = 0 and Boys = 1, ^b^ Children = 0 and Adolescents = 1, ^c^ Muslim = 0 and Non-Muslim = 1, ^d^ Rural = 0 and Urban = 1, ^e^ Public = 0 and Private = 1.

## Data Availability

The corresponding author can provide the data used in this work upon reasonable request.
